# Developing an Enzyme-Assisted Derivatization Method for Analysis of C_27_ Bile Alcohols and Acids by Electrospray Ionization-Mass Spectrometry

**DOI:** 10.3390/molecules24030597

**Published:** 2019-02-07

**Authors:** Jonas Abdel-Khalik, Peter J. Crick, Eylan Yutuc, Yuqin Wang, William J. Griffiths

**Affiliations:** Swansea University Medical School, ILS1 Building, Swansea University, Singleton Park, Swansea SA2 8PP, Wales, UK; jonas.abdelkhalik@gmail.com (J.A.-K.); peter.crick@gmail.com (P.J.C.); eylan.yutuc@swansea.ac.uk (E.Y.); y.wang@swansea.ac.uk (Y.W.)

**Keywords:** bile alcohol, cholestanoic acid, oxysterol, sterolomics, enzyme-assisted derivatization, electrospray ionization-mass spectrometry, Girard reagent

## Abstract

Enzyme-assisted derivatization for sterol analysis (EADSA) is a technology designed to enhance sensitivity and specificity for sterol analysis using electrospray ionization–mass spectrometry. To date it has only been exploited on sterols with a 3β-hydroxy-5-ene or 3β-hydroxy-5α-hydrogen structure, using bacterial cholesterol oxidase enzyme to convert the 3β-hydroxy group to a 3-oxo group for subsequent derivatization with the positively charged Girard hydrazine reagents, or on substrates with a native oxo group. Here we describe an extension of the technology by substituting 3α-hydroxysteroid dehydrogenase (3α-HSD) for cholesterol oxidase, making the method applicable to sterols with a 3α-hydroxy-5β-hydrogen structure. The 3α-HSD enzyme works efficiently on bile alcohols and bile acids with this stereochemistry. However, as found by others, derivatization of the resultant 3-oxo group with a hydrazine reagent does not go to completion in the absence of a conjugating double bond in the sterol structure. Nevertheless, Girard P derivatives of bile alcohols and C_27_ acids give an intense molecular ion ([M]^+^) upon electrospray ionization and informative fragmentation spectra. The method shows promise for analysis of bile alcohols and 3α-hydroxy-5β-C_27_-acids, enhancing the range of sterols that can be analyzed at high sensitivity in sterolomic studies.

## 1. Introduction

Sterols represent one of the major classes of lipids found in living systems [1]. In mammals, cholesterol represents the archetypal sterol. It is metabolized through a myriad of intermediates to C_21–18_ steroids and to C_24_ bile acids [2,3,4,5,6,7,8,9]. For decades there was little interest in these intermediates, however, in recent years the situation has changed with the realization that intermediates in bile acid biosynthesis are ligands to nuclear receptors, including the liver X receptors (LXRs, NR1H3, NR1H2) [10,11,12,13], farnesoid X receptor (FXR, NR1H4) [14], pregnane X receptor (PXR, also known as xenobiotic sensing nuclear receptor, SXR, NR1I2) [15,16], RAR-related orphan receptor γt (RORγt, NR1F3) [17], and estrogen receptors (ERs, NR3A1, NR3A2) [18]. They are also related to G protein-coupled receptors (e.g., Epstein-Barr virus induced gene 2 (EBI2, GPR183) [19,20] and smoothened (SMO, FZD11) [21,22]), and are involved in the regulation of cholesterol biosynthesis by binding to INSIG1 (insulin induced gene 1) [23]. Cholesterol metabolites have traditionally been analyzed by gas-chromatography-mass spectrometry (GC-MS) [3,4,6,24,25], however, liquid chromatography (LC)-MS, is currently taking a dominant role in their analysis [5,26].

Analysis of cholesterol metabolites is valuable for the diagnosis of rare inborn errors of metabolism, and defects in bile acid and steroid synthesizing enzymes are efficiently characterized by LC-MS or GC-MS analysis of plasma or urine [6,27,28,29]. By performing these analyses, unexpected metabolites are identified, which are normally only minor components of the sterolome, but which are abundant in the disease state [7,8,9]. When these unexpected metabolites are considered, it becomes evident that the complexity of the sterolome is enormous. Sterolomics is one of the subdivisions of lipidomics, however, in most lipidomic studies, sterols are underrepresented; this is because, other than cholesterol and its esters, they are not abundant, and their ionization characteristics in positive-ion electrospray ionization (ESI)-MS (the dominant ionization method in lipidomics) are poor [30,31]. To improve the sensitivity for sterol analysis, many groups adopt a derivatization strategy where sterols are chemically modified to improve their ionization characteristics [32,33,34,35,36,37,38,39].

**Scheme 1:**
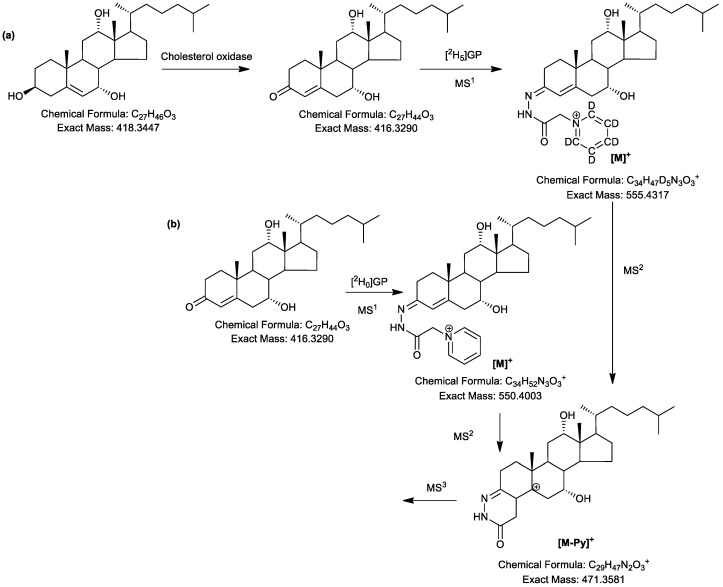

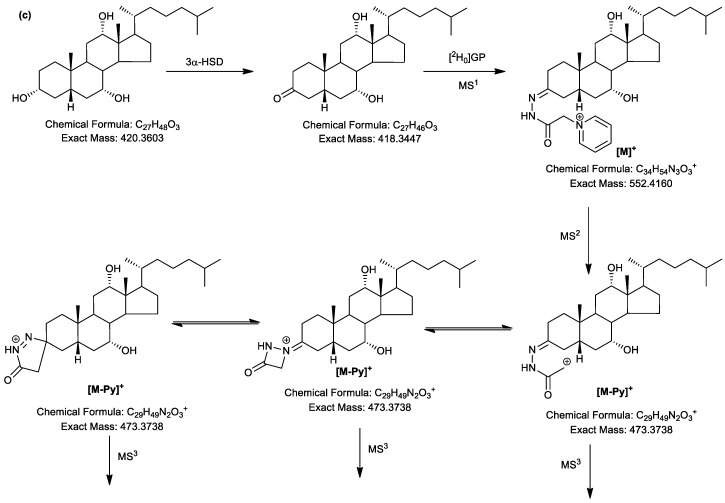
Enzyme-assisted derivatization for sterol analysis (EASDA): (**a**) EADSA of the 3β-hydroxy-5-ene function using cholesterol oxidase and [^2^H_5_] Girard P reagent ([^2^H_5_]GP); (**b**) derivatization of the 3-oxo-4-ene function with [^2^H_0_]GP; (**c**) EADSA of the 3α-hydroxy-5β-hydrogen function with 3α-hydroxysteroid dehydrogenase (3α-HSD) and [^2^H_0_]GP. Derivatives with the [^2^H_5_]GP and [^2^H_0_]GP reagents can be combined and analyzed in a single LC-MS run. Other isotope-coded GP reagents have been synthesized to allow triplex analysis if required [45].

One derivatization strategy that has lately become popular is enzyme-assisted derivatization for sterol analysis (EADSA, Scheme 1) [8,9,12,13,17,40,41,42,43,44,45,46,47]. EADSA technology was designed to add specificity and sensitivity to sterol analysis [40,44,45,46]. This is achieved by specifically targeting the 3β-hydroxy-5-ene or 3β-hydroxy-5α-hydrogen function in sterols and converting the 3β-hydroxy to a 3-oxo group with bacterial cholesterol oxidase from *Brevibacterium* or *Streptomyces* sp. [40,41]. Once introduced, the 3-oxo group is derivatized with the positively charged Girard hydrazine reagent, introducing a charge-tag to the target analyte and improving sensitivity in ESI-MS. A limitation of the existing protocol is that it is not applicable to sterols with a 3α-hydroxy group. Here we describe how this limitation is overcome for the analysis of bile alcohols and C_27_ bile acids with this stereochemistry. The methodology is potentially applicable for C_24_ bile acids but requires further optimization to achieve similar sensitivity as for C_27_ alcohols and acids.

## 2. Results

### 2.1. Oxidation Efficiency of 3α-Hydroxysteroid Dehydrogenase (3α-HSD)

The efficiency of oxidation of 3α-HSD towards 3α-hydroxy-5β-substrates was evaluated using cholic acid (3α,7α,12α-trihydroxy-5β-cholan-24-oic acid, BA-3α,7α,12α-triol) and 3α,7α,12α-trihydroxy-5β-cholestan-26-oic acid (CA-3α,7α,12α-triol) as representative analytes, because unlike neutral sterols, C_24_ and C_27_ bile acids are readily ionized by ESI (negative-ion mode) and can be detected in LC-MS analysis in both their 3α-hydroxy or 3-oxo forms. To determine oxidization efficiency, the amounts of unoxidized (3α-hydroxy) and oxidized (3-oxo) acids were determined after different incubation periods and incorporated into Equation (1).

Oxidation efficiency (%) = amount oxidized/(amount oxidized + amount unoxidized) × 100%(1)

Using similar reaction conditions to those employed in the current study (see Section 4.2.1), Une et al. have shown that an incubation time of 20 h leads to about 95% conversion of the 3α-hydroxy group to the 3-one in cholic acid and in most bile alcohols [48]. We found that reducing the incubation time to 14 h gave a similarly high yield (>95%) of product for CA-3α,7α,12α-triol and cholic acid. Either incubation time, 14 h or 20 h, gave satisfactory results. In the current study, similarly high efficiencies of oxidation (>95%) were found for both the glycine and taurine conjugates of cholic acid and for unconjugated chenodeoxycholic (3α,7α-dihydroxy-5β-cholan-24-oic), deoxycholic (3α,12α-dihydroxy-5β-cholan-24-oic) and lithocholic (3α-hydroxy-5β-cholan-24-oic) acids.

### 2.2. Girard P (GP)-Derivatisation of 3-Oxo Groups

Previous studies have shown that hydrazone formation is very efficient towards 3-oxo-4-ene substrates and to other α,β-unsaturated ketones [40,41,49,50]. In earlier EADSA studies using cholesterol oxidase in phosphate buffer to convert 3β-hydroxy-5-ene sterols to their 3-oxo-4-ene equivalents, GP derivatization was achieved by simply adding methanol to the incubation solution to give a 70% methanol solution and then adding acetic acid and GP hydrazine reagent [40,41,45,46,50,51,52]. However, the buffer required for 3α-HSD oxidation of the 3α-hydroxy group to the 3-one is 100 mM pyrophosphate buffer, pH 8.9, and upon methanol addition, necessary for subsequent hydrazone formation, a precipitate is formed. This can be avoided by limiting the methanol content to 5%, however, under these conditions hydrazone formation is reversed back to the hydrazine and free carbonyl. For this reason, following incubation with 3α-HSD, samples were desalted on an Oasis HLB reversed-phase column and eluted in methanol, a solvent suitable for subsequent GP hydrazone formation. The GP derivatization efficiency was assessed by LC-MS in the negative-ion mode by comparing the amount of underivatized oxidized acid present before and after the GP derivatization step and incorporating the data into Equation (2).

Derivatization efficiency (%) = 100% − [(amount underivatized acid after derivatization/amount underivatized acid before derivatization) × 100%](2)

Unlike 3-oxo-4-ene sterols, which are derivatized with 100% efficiency in acidic methanol [40], the derivatization efficiency for the 3-oxo-5β-hydrogen compounds formed from their 3α-hydroxy-5β-hydrogen substrates (cholic, chenodeoxycholic, deoxycholic acids and CA-3α,7α,12α-triol), was only 45–60%. Taurine- and glycine-conjugated cholic acid gave a similar degree of derivatization efficiency after 3α-HSD oxidation. Despite the moderate yield of derivatization products, the high sensitivity provided by GP derivatization of unconjugated substrates (see Section 2.3.) negates this imperfection.

### 2.3. LC-MS Analysis of Oxidised/Derivatised 3α-Hydroxy-5β-Hydrogen Substrates

In this preliminary study we have not optimized the chromatographic or MS conditions for the GP-derivatized target compounds but rather used existing LC-MS conditions used previously for GP-derivatized sterols [44,45,46,52]. The logic behind this is that by using isotope-labelled GP reagent, the ultimate aim will be to analyze sterols oxidized with cholesterol oxidase or 3α-HSD in a single LC-MS run. Neither have we performed detailed investigations of limits of quantification or linearity of dynamic range in the current study. However, we find that the sensitivity obtained here for 3α-HSD oxidized/GP-derivatized C_27_ sterols with a 3α-hydroxy-5β-stereochemistry is of the same order of magnitude to that obtained for GP derivatives generated after cholesterol oxidase treatment of 3β-hydroxy-5-ene substrates. For the C_27_ substrates an on-column limit of detection (LOD, signal/noise, 5:1) of 250 fg was achieved. The on-column LOD for the C_24_ acid, cholic acid, was 250 pg. More work is required to explain this discrepancy in sensitivity and the even poorer sensitivity with glycine- and taurine-conjugated acids. Optimization of the ion-source conditions for different groups of analytes, or at least compromise in the settings chosen, is likely to be necessary.

### 2.4. MS^n^ Fragmentation

A major driver for the current study was the poor fragmentation properties of unconjugated C_24_ and C_27_ bile acids under conditions of ESI–tandem MS (MS/MS) at low collision energy (<100 eV) [5,53,54,55,56] (see also MassBank of North America http://mona.fiehnlab.ucdavis.edu/). This has led to many studies in which the precursor ion at unit mass resolution is also used as the “product ion” for generation of LC–multiple reaction monitoring (MRM) chromatograms. Once derivatized with the GP reagent, both bile acids and bile alcohols fragment under low-energy conditions with the loss of the pyridine group, resulting in [M-Py]^+^ ions (see Scheme 1). These ions can be fragmented further in ion-trap instruments to give multistage fragmentation (MS^3^, [M]^+^→[M-Py]^+^→) spectra rich in fragment ions. The advantage of MS^3^ is that it provides an extra dimension of separation compared to MS^2^, where spectra are a composite of fragment ions derived from desired and undesired coselected precursor ions.

#### 2.4.1. Triols and Tetrols

**Figure 1:**
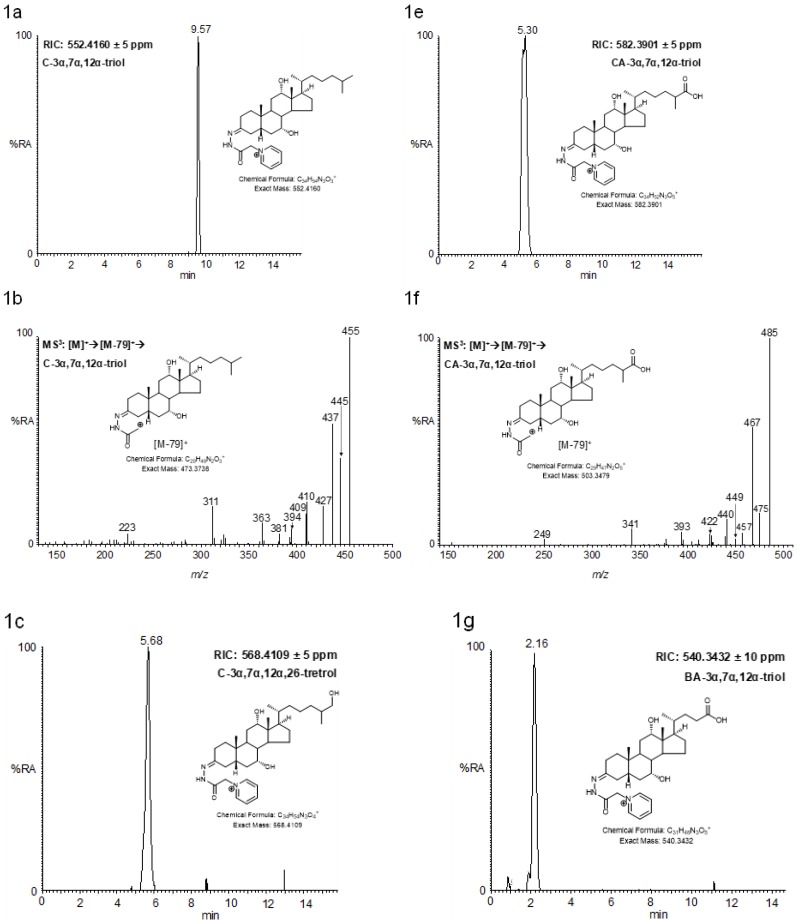

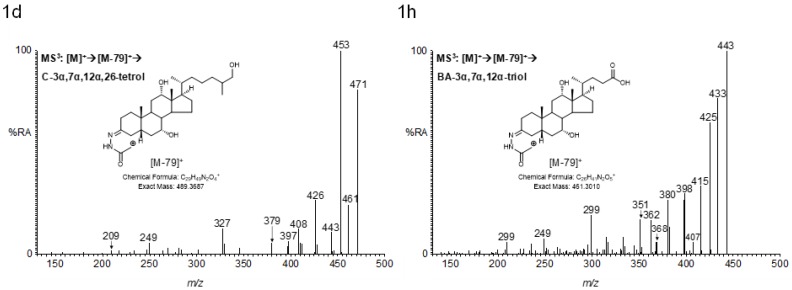
Reconstructed-ion chromatograms (RICs) and multistage fragmentation (MS^3^) ([M]^+^→[M-Py]^+^→) spectra of oxidized/GP-derivatized 3α-hydroxy-5β-bile alcohols and acids: (**a**,**b**) C-3α,7α,12α-triol; (**c**,**d**) C-3α,7α,12α,26-tetrol; (**e**,**f**) CA-3α,7α,12α-triol; (**g**,**h**) cholic acid. The RICs were generated from mass spectra recorded in the Orbitrap mass analyzer at a resolution of 120,000 (FWHM definition at *m*/*z* 400), with an *m*/*z* window of ± 5 ppm. MS^3^ spectra were generated in the linear ion-trap and recorded at the ion-trap detector of the Orbitrap-Elite mass spectrometer. Mass accuracy for fragment ion measurements made with the linear ion-trap is typically ± 0.3 Da. Postulated compositions of fragment ions are listed in Table 1. Note that the data for cholic acid was generated on an earlier version of instrument (i.e., Orbitrap-LTQ) at lower resolution and with reduced mass accuracy.

Shown in Figure 1 are representative reconstructed-ion chromatograms (RICs) and MS^3^ ([M]^+^→[M-Py]^+^→) spectra of oxidized/GP-derivatized C_27_ bile alcohols 5β-cholestane-3α,7α,12α-triol (C-3α,7α,12α-triol) and 5β-cholestane-3α,7α,12α,26-tetrol (C-3α,7α,12α,26-tetrol)), the C_27_ acid (CA-3α,7α,12α-triol), and the C_24_ trihydroxy bile acid (cholic acid).

**Scheme 2:**
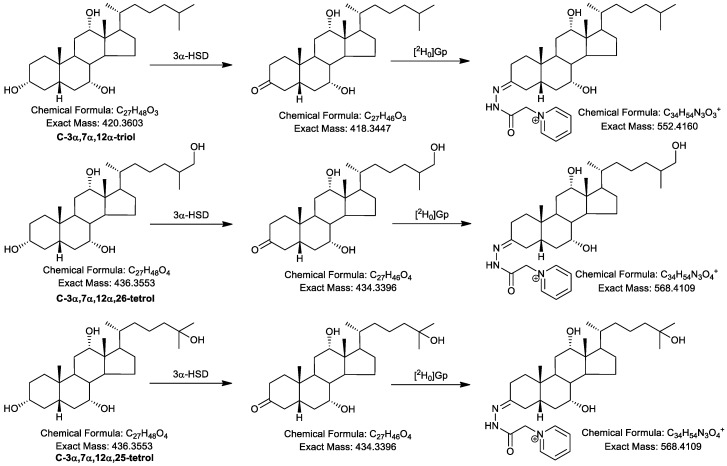

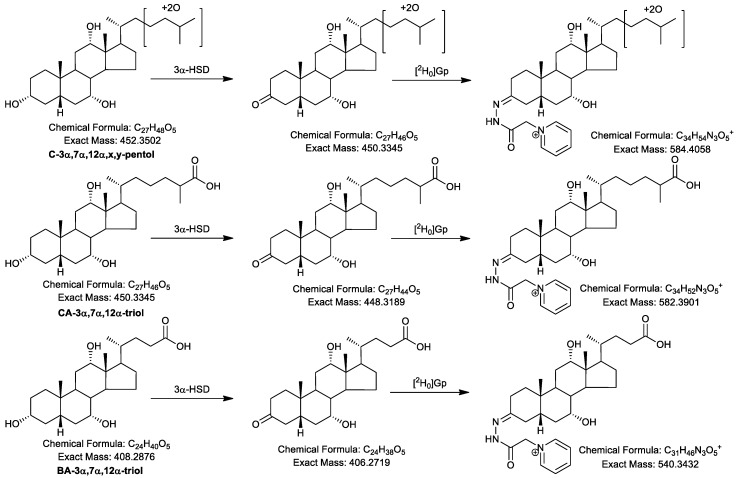
Structures of bile alcohols and acids, and their products of 3α-HSD oxidation and GP-derivatization.

**Scheme 3:**
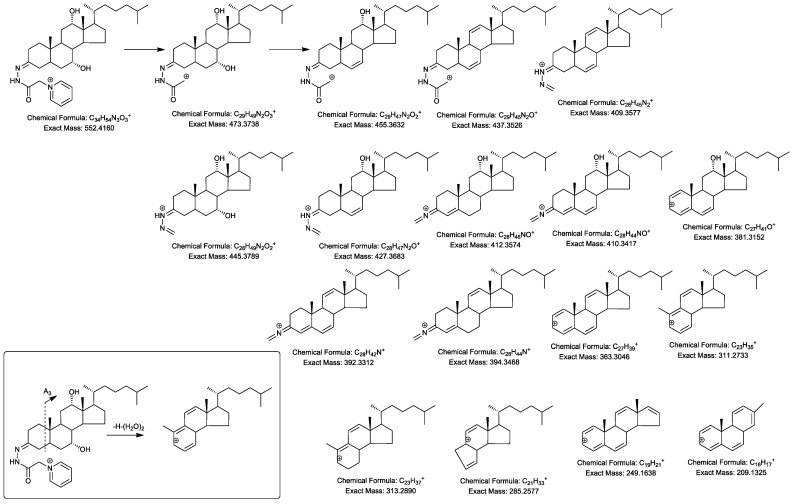
MS^2^ and MS^3^ fragmentation of oxidized/GP derivatized bile alcohols as illustrated by C-3α,7α,12α-triol. For simplicity the fragmented Girard derivatizing group is shown in its linear isomeric form. The inset shows fragmentation route A_3_ leading to the [A_3_-H-(H_2_O)_2_]^+^ fragment ion. Cyclic isomers are depicted in Scheme 1. See Supplemental Schemes S1–S4 for fragmentation schemes of other trihydroxy- and tetrahydroxy-bile alcohols and trihydroxy-bile acids. Table 1 correlates *m*/*z* with fragment ion composition.

The MS^3^ spectra show considerable similarity, with many fragment ions in the spectrum of the C_26_ acid and tetrol being shifted by *m*/*z* 30 and *m*/*z* 16, respectively from the corresponding triol. This is explained by the introduction of a carboxylic acid group (+ O_2_ − H_2_) or hydroxy (+ O) group to the terminal carbon (C-26) of the sterol side-chain (Scheme 2). Postulated structures of fragment ions for C-3α,7α,12α-triol are shown in Scheme 3 and for C-3α,7α,12α,26-tetrol and CA-3α,7α,12α-triol in Supplemental Schemes S1 and S2, and are listed in Table 1.

**Figure 2:**
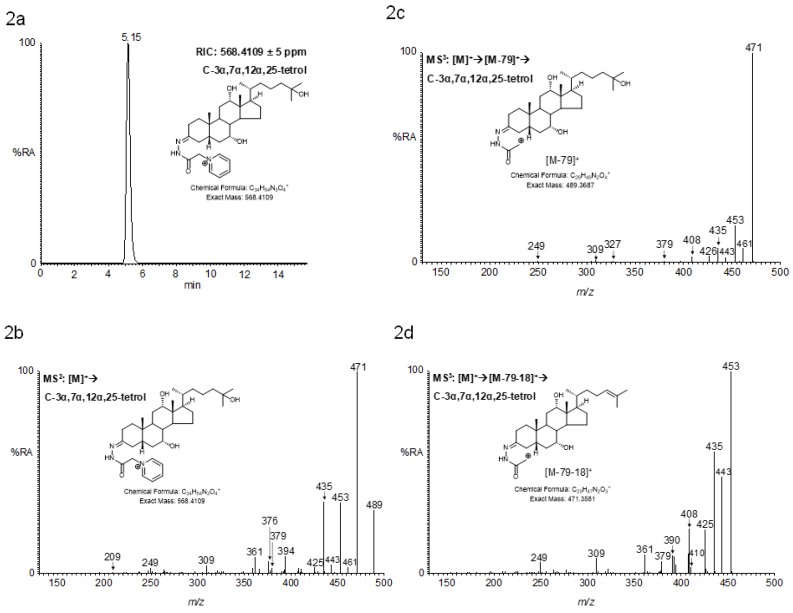
(**a**) RIC and (**b**) MS^2^ ([M]^+^→), (**c**) MS^3^ ([M]^+^→[M-Py]^+^→), (**d**) ([M]^+^→[M-Py-18]^+^→) spectra of oxidized/GP-derivatized C-3α,7α,12α,25-tetrol. For comparison, MS^2^ ([M]^+^→) spectra of C-3α,7α,12α-triol and C-3α,7α,12α,26-tetrol and the MS^3^ ([M]^+^→[M-Py-18]^+^→) spectrum of C-3α,7α,12α,26-tetrol are shown in Supplemental Figure S1a–c respectively. Mass spectra recorded at the peak of the RIC for these and other sterols analyzed are shown in Supplemental Figure S2. Data was generated on the Orbitrap-Elite mass spectrometer as in Figure 1. See Table 1 to correlate *m*/*z* with fragment ion composition.

When a hydroxy group is positioned at C-25 rather than C-26, the lability of the hydroxy group leads to a more intense [M-Py-18]^+^ than [M-Py]^+^ ion in the MS^2^ ([M]^+^→) spectrum of 5β-cholestane-3α,7α,12α,25-tetrol (C-3α,7α,12α,25-tetrol, Figure 2b) than in its epimer C-3α,7α,12α,26-tetrol (Supplemental Figure S1b). See Table 1 to correlate *m*/*z* with fragment ion composition. The MS^3^ ([M]^+^→[M-Py-18]^+^→) spectrum of C-3α,7α,12α,25-tetrol (Figure 2d) is almost identical to the MS^3^ ([M]^+^→[M-Py]^+^→) of C-3α,7α,12α-triol (Figure 1b) but with an offset of *m*/*z* -2 (+ O – H_2_O, see also Supplemental Scheme S3).

Unsurprisingly, the MS^3^ ([M^+^→[M-Py]^+^→) spectrum of the C_24_ acid, cholic acid (Figure 1h), shows the same pattern of fragment ions as the C_27_ acid CA-3α,7α,12α-triol (Figure 1f) but offset by *m*/*z* -42 (-C_3_H_6_), corresponding to the mass difference between equivalent C_27_ and C_24_ acids (cf. Supplemental Schemes S2 and S4).

**Figure 3:**
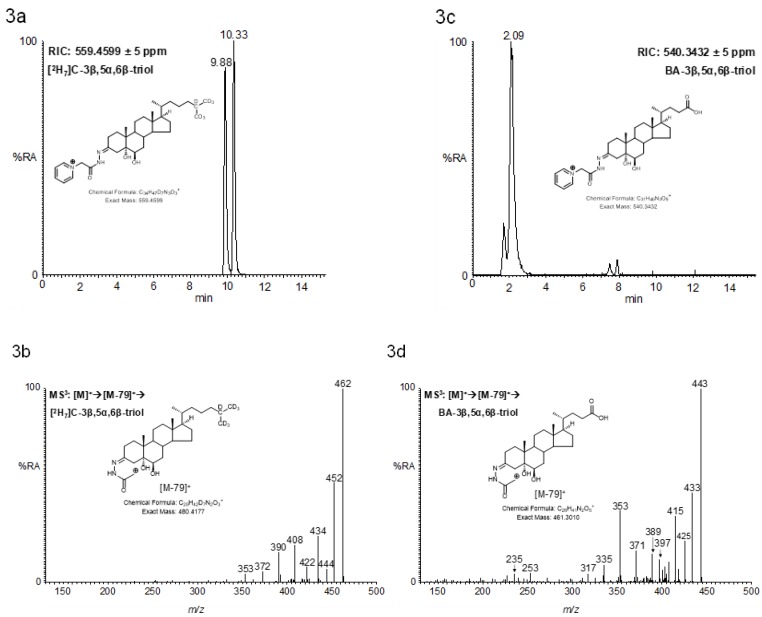
RICs and MS^3^ ([M]^+^→[M-Py]^+^→) spectra of cholesterol oxidase-oxidized/GP-derivatized 3β,5α,6β-triols: (**a**,**b**) [25,26,26,26,27,27,27-^2^H_7_]cholestane-3β,5α,6β-triol ([^2^H_7_]C-3β,5α,6β-triol); (**c**,**d**) 3β,5α,6β-trihydroxycholanoic acid (BA-3β,5α,6β-triol). See reference [8] for a description of fragmentation pathways. GP-derivatized sterols can give syn and anti conformers resulting in twin chromatographic peaks which may or may not be resolved. Data was generated on the Orbitrap-Elite mass spectrometer as in Figure 1.

A key structurally distinct fragment ion for all the 3α,7α,12α-triols is the [A_3_-H-(H_2_O)_2_]^+^ ion (or [A_3_-H-(H_2_O)_3_]^+^ when an additional hydroxy group is at C-25, Table 1), a triply unsaturated carbonium ion consisting of B-, C- and D-rings plus the C_17_ side-chain, where charge is delocalized across the three double bonds in the ring system (Scheme 3, inset). An equivalent fragment ion is not observed in cholesterol oxidase-oxidized/GP-derivatized 3β,5α,6β-triols (Figure 3).

#### 2.4.2. Pentols

The human autosomal recessive disease, cerebrotendinous xanthomatosis (CTX), results from a deficiency in cytochrome P450 27A1 (CYP27A1) [57], a key enzyme in the conversion of cholesterol to bile acids [28,29]. As a consequence of this deficiency, polyhydroxy-bile alcohols are produced [58,59], providing an alternative route for bile acid biosynthesis and cholesterol removal [60].

**Figure 4:**
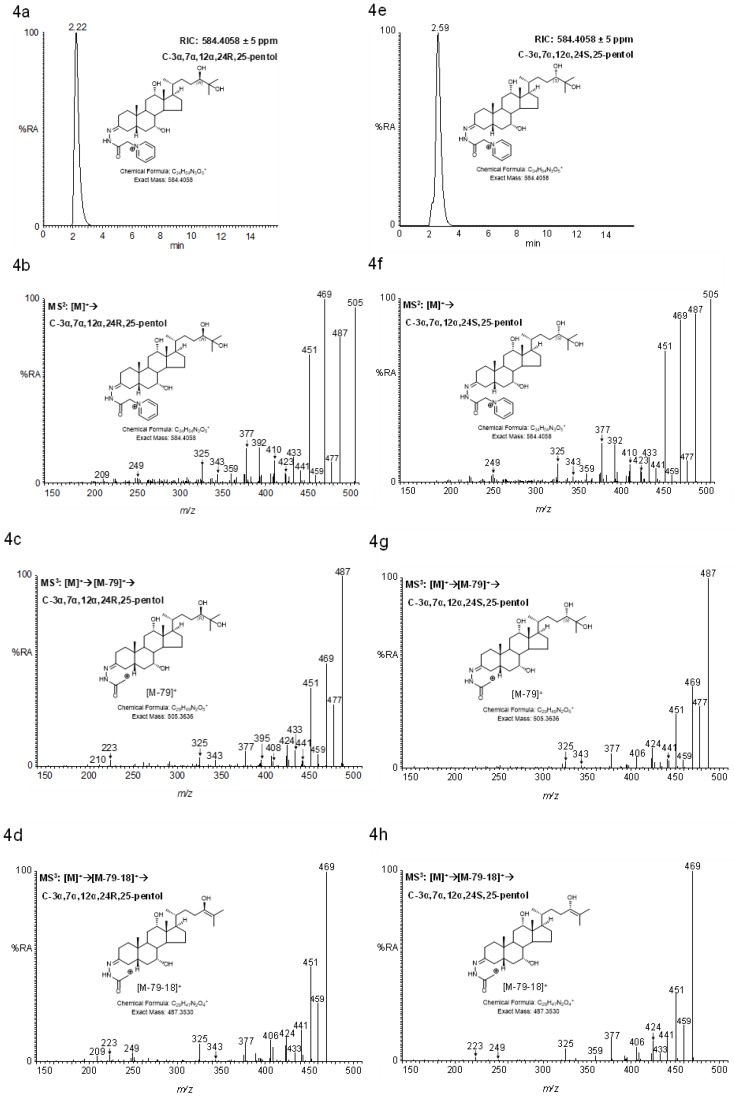
LC–(MS)MS^n^ analysis of oxidized/GP-derivatized C-3α,7α,12α,24R,25-pentol and C-3α,7α,12α,24S,25-pentol: (**a**) RIC, (**b**) MS^2^ ([M]^+^→), (**c**) MS^3^ ([M]^+^→[M-Py]^+^→), and (**d**) ([M]^+^→[M-Py-18]^+^→) from the analysis of C-3α,7α,12α,24R,25-pentol. (**e**) RIC, (**f**) MS^2^ ([M]^+^→), (**g**) MS^3^ ([M]^+^→[M-Py]^+^→), and (**h**) ([M]^+^→[M-Py-18]^+^→) from the analysis of C-3α,7α,12α,24S,25-pentol. Data was generated on the Orbitrap-Elite mass spectrometer as in Figure 1. Fragment ions are described in Table 1 and postulated structures are shown in Scheme 4.

As with C-3α,7α,12α,25-tetrol, the presence of a labile 25-hydroxy group in the epimers 5β-cholestane-3α,7α,12α,24R,25-pentol (C-3α,7α,12α,24R,25-pentol) and 5β-cholestane-3α,7α,12α,24S,25-pentol (C-3α,7α,12α,24S,25-pentol) results in abundant [M-Py-18]^+^ ions in the MS^2^ ([M]^+^→) spectra, and the MS^3^ ([M]^+^→[M-Py-18]^+^→) spectra resembles the MS^3^ ([M]^+^→[M-Py]^+^→) spectra of cholestanetetrols but is offset by *m*/*z* -2 (+ O – H_2_O) (Figure 4). Although the MS^n^ spectra are very similar, the two epimers are readily separated on the LC column. Here in the MS^2^ ([M]^+^→) and MS^3^ ([M]^+^→[M-Py]^+^) spectra, in addition to the [A_3_-H-(H_2_O)_2_]^+^ fragment ion, a [A_3_-H-(H_2_O)_3_]^+^ fragment ion is also prominent (See Table 1 to correlate *m*/*z* with fragment ion composition). 

**Figure 5:**
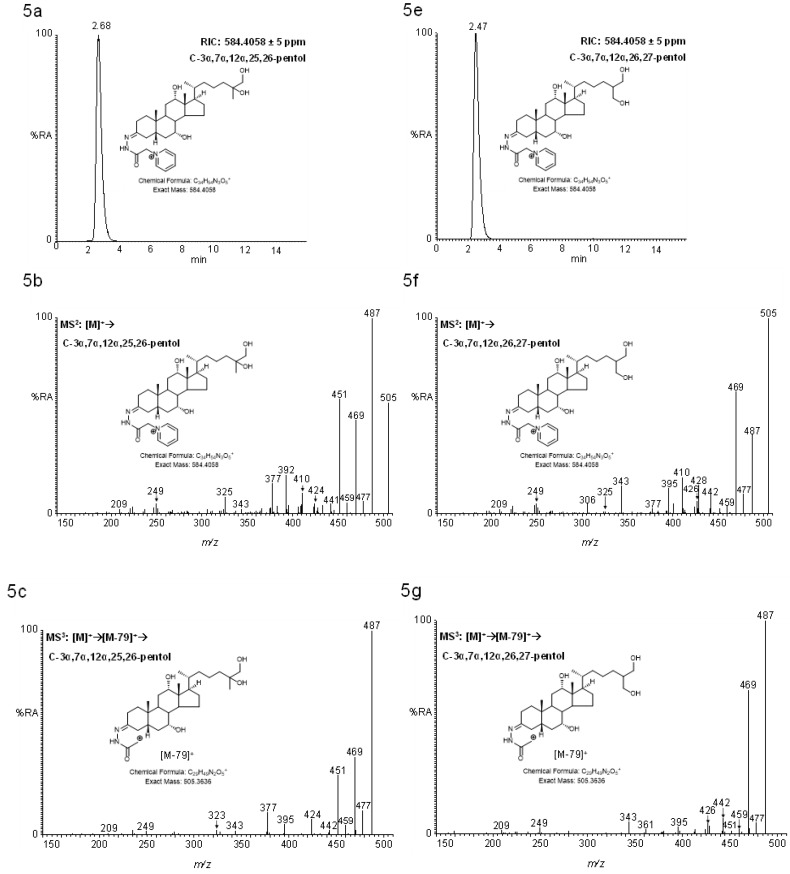

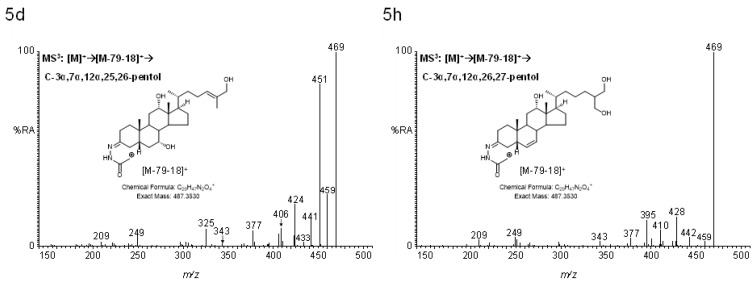
LC–(MS)MS^n^ analysis of oxidized/GP-derivatized C-3α,7α,12α,25,26-pentol and C-3α,7α,12α,26,27-pentol: (**a**) RIC, (**b**) MS^2^ ([M]^+^→), (**c**) MS^3^ ([M]^+^→[M-Py]^+^→), and (**d**) ([M]^+^→[M-Py-18]^+^→) from the analysis of C-3α,7α,12α,25,26-pentol. (**e**) RIC, (**f**) MS^2^ ([M]^+^→), (**g**) MS^3^ ([M]^+^→[M-Py]^+^→), and (**h**) ([M]^+^→[M-Py-18]^+^→) from the analysis of C-3α,7α,12α,26,27-pentol. Data was generated on the Orbitrap-Elite mass spectrometer as in Figure 1. Fragment ions are described in Table 1 and postulated structures are shown in Supplemental Schemes S5 and S6.

Movement of the hydroxy group from C-24 to C-26 as in 5β-cholestane-3α,7α,12α,25,26-pentol (C-3α,7α,12α,25,26-pentol) results in a small delay in retention time and subtle changes to the MS^2^ ([M]^+^→) and MS^3^ ([M]^+^→[M-Py]^+^→ and [M]^+^→[M-Py-18]^+^→) spectra, for example, reduced abundance of fragment ions having lost four water molecules compared the equivalent having lost three water molecules (i.e., [M-79-36-36]^+^/[M-79-36-18]^+^) (Figure 5b–d, cf. Figure 4b–d,f–h). The bile alcohol 5β-cholestane-3α,7α,12α,26,27-pentol (C-3α,7α,12α,26,27-pentol) elutes between the 24R- and 25S-epimers of C-3α,7α,12α,24,25-pentol, but gives very different MS^n^ spectra to the other cholestanepentols on account of the absence of a labile C-25-hydroxy group (Figure 5f–h, Supplemental Scheme S6). This is reflected in the ratio of fragment ions [M-79-36-18]^+^/[M-79-36]^+^ which is greatly reduced compared to pentols with a 25-hydroxy group. The comparative stability of the primary hydroxy groups at the termini, C-26 and C-27, is further reflected by an absence of fragment ions having lost four water molecules (e.g., [M-79-36-36]^+^).

**Figure 6:**
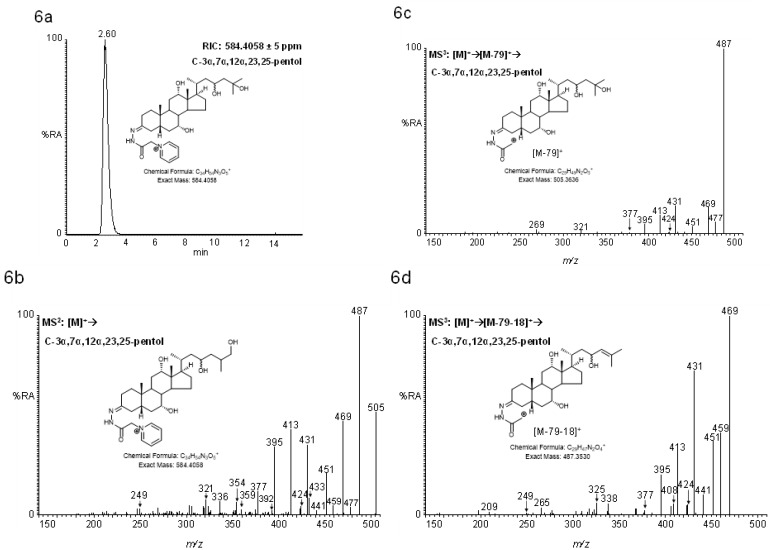
LC–(MS)MS^n^ analysis of oxidized/GP-derivatized C-3α,7α,12α,23,25-pentol. (**a**) RIC, (**b**) MS^2^ ([M]^+^→), (**c**) MS^3^ ([M]^+^→[M-Py]^+^→), and (**d**) ([M]^+^→[M-Py-18]^+^→). Data was generated on the Orbitrap-Elite mass spectrometer as in Figure 1. Fragment ions are described in Table 1 and postulated structures are shown in Supplemental Schemes S7.

Although it co-elutes with C-3α,7α,12α,24S,25-pentol, 5β-cholestane-3α,7α,12α,23,25-pentol (C-3α,7α,12α,23,25-pentol) gives unique MS^n^ spectra (Figure 6). Each of the MS^2^ ([M]^+^→), MS^3^ ([M]^+^→[M-Py]^+^→) and MS^3^ ([M]^+^→[M-Py-18]^+^→) spectra show an unusual pattern of fragment ions at *m*/*z* 431.3, 413.3, and 395.3. It is not immediately obvious why this triad of fragment ions is so distinct for this molecule. Neither are the structures or chemical compositions of all these fragments easy to reconcile with the MS^3^ ([M]^+^→[M-Py-18]^+^→) spectrum.

## 3. Discussion

In this communication we describe preliminary studies to develop an enzyme-assisted derivatization for C_27_ bile alcohols and acids with a 3α-hydroxy-5β-hydrogen stereochemistry. The method still requires further optimization, particularly with respect to the GP-derivatization step which only gave a 45%–60% yield. Despite this, the considerable sensitivity of GP-derivatives makes the moderate yield tolerable. Although the LC-MS sensitivity for detection of C_24_ acids was not as good as for C_27_ acids, the rich MS^3^ fragment ion spectra provide a significant advantage over conventional MS/MS spectra of unconjugated acids where few fragment ions are observed. The on-column detection limit of 250 fg for C_27_ analytes translates to a limit of detection of about 0.2 ng/mL if 100 µL of biological fluid is worked up and 1% injected on-column, as in our usual procedure with EADSA [52]. For comparison, Johnson et al. could measure CA-3α,7α,12α-triol, after derivatization to the dimethylaminoethyl ester, at a concentration of about 60 ng/mL in as little as 5 µL of plasma, with 20% injected-on column [38], while DeBarber et al. determined the limit of quantification of 7α,12α-dihydroxy-5β-cholestan-3-one, the 3-oxo form of C-3α,7α,12α-triol, to be 20 ng/mL from 4 µL of plasma after derivatization to the oxime with (*O*-(3-trimethylammoniumpropyl) hydroxylamine) bromide [39]. We have not yet rigorously tested the repeatability of the EADSA methodology in biological samples. This will become relevant with the availability of isotope-labelled standards, which can be synthesized by methods described by Johnson et al. and by Shoda et al. [61]. Isotope-labelled internal standards will similarly facilitate the progression of the method to a quantitative format. We did not attempt to optimize LC-MS conditions for the GP-derivatives analyzed in this study; instead we used previously optimized conditions for derivatized oxysterols. The logic behind this was to allow the expansion of our sterol profiling method to include bile acids and alcohols derivatized with [^2^H_0_]GP after 3α-HSD treatment and oxysterols, and cholestenoic and cholenoic acids derivatized with [^2^H_5_]GP after cholesterol oxidase treatment, or vice versa, in a single LC-MS run. At present there are challenges with this strategy, as efficient ionization of glycine- and taurine-conjugated bile acids requires different ion-source conditions from the unconjugated GP-derivatives. 

## 4. Materials and Methods

### 4.1. Materials

CA-3α,7α,12α-triol (LMST04030001) and [^2^H_7_]C-3β,5α,6β-triol were from Avanti Polar Lipids (Alabaster, AL, USA). Bile alcohols, C-3α,7α,12α-triol (LMST04030035), C-3α,7α,12α,25-tetrol (LMST04030037) and C-3α,7α,12α,26-tetrol, (LMST04030159 or LMST04030160), C-3α,7α,12α,24R,25-pentol (LMST04030177), C-3α,7α,12α,24S,25-pentol (LMST04030039), C-3α,7α,12α,25,26-pentol (LMST04030016), 3α,7α,12α,26,27-pentol (LMST04030041) and C-3α,7α,12α,23,25-pentol (LMST01010240 or LMST01010241) were kind gifts from Professor Jan Sjӧvall, Karolinska Institutet, Stockholm. BA-3β,5α,6β-triol (LMST04010339) was a kind gift from Professor Douglas Covey, Washington University. Other C_24_ bile acids were from Sigma-Aldrich (Dorset, UK) or Fluka Chemie (Buchs, Switzerland). 3α-Hydroxysteroid dehydrogenase (3α-HSD) from *Pseudomonas testosteroni* was from Sigma-Aldrich (Dorset, UK). β-Nicotinamide adenine dinucleotide hydrate and sodium pyrophosphate decahydrate were from Sigma-Aldrich. [^2^H_0_]GP ([1-(carboxymethyl)pyridinium chloride hydrazide]) reagent was from TCI Europe (Oxford, UK). [^2^H_5_]GP reagent was synthesized as the bromide salt as described in Crick et al. [45]. Solid phase extraction (SPE) columns, certified Sep-Pak C_18_, 200 mg (3 cm^3^), and 60 mg Oasis HLB (3 cm^3^), were from Waters Inc. (Elstree, UK). Solvents were obtained from Fisher Scientific (Loughborough, UK). Acetic acid and formic acid were of AnalaR NORMAPUR grade (BDH, VWR, Lutterworth, UK).

### 4.2. Methods

#### 4.2.1. Oxidation and Derivatization

Oxidation of analytes by 3α-HSD was essentially as described by Une et al. [48]. β-NAD^+^ hydrate (19.8 mg) was dissolved in 100 mM pyrophosphate buffer pH 8.9 (1 mL). Analyte (40–400 ng) dissolved in ethanol (10 µL) was added to the buffered solution giving a final concentration of 1% ethanol, followed by addition of 3α-HSD (0.06 units). After incubation at room temperature for 20 h, methanol (40 µL) was added (giving an organic content of 5%). To separate oxidized analyte from buffer, the solution was loaded onto a HLB column (60 mg, previously washed with methanol, 6 mL, and conditioned with 5% methanol, 6 mL) followed by a rinse with 5% methanol (0.5 mL). The column was then further washed with 5% methanol (6 mL). Analytes were eluted with methanol (2 mL). For samples to be analyzed by ESI in the negative-ion mode, to monitor oxidation efficiency, the methanol eluate was diluted with water to give a 60% methanol solution and was analyzed by LC-MS on the Orbitrap-Elite high resolution mass spectrometer (Thermo Fisher Scientific, Waltham, MA, USA) at 120,000 resolution (full width at half maximum height at *m*/*z* 400). To derivatize samples with GP reagent, glacial acetic acid (150 µL) was added followed by GP reagent (150 mg chloride salt, 190 mg bromide salt) and the mixture was left at room temperature overnight. The next day, water (1 mL) was added immediately prior to a second SPE step. This second SPE step was performed with recycling on an Oasis HLB column (60 mg) to remove excess derivatization reagent and was carried out as described in Abdel-Khalik et al. [52].

#### 4.2.2. LC-MS(MS^n^) Analysis

LC-MS(MS^n^) was performed in the positive-ion mode as described in Abdel-Khalik et al. utilizing the Orbitrap-Elite hybrid MS preceded by a Dionex Ultimate 3000 LC system (Dionex, now Thermo Fisher Scientific) [52]. For analysis of underivatized acids in the negative-ion mode, other than for polarity reversal and a change of column from a Hypersil Gold C_18_ to a Kinetex core-shell technology XB-C_18_ column (2.6 µm, 2.1 mm × 50 mm, Phenomenex, Macclesfield, UK), the method was as for positive-ion mode LC-ESI-MS(MS^n^) as described in Abdel-Khalik et al. [52].

## 5. Patents

The derivatization method described in this manuscript is patented by Swansea University (US9851368B2) and licensed by Swansea Innovations to Avanti Polar Lipids and to Cayman Chemical Company.

## Figures and Tables

**Scheme 4:**
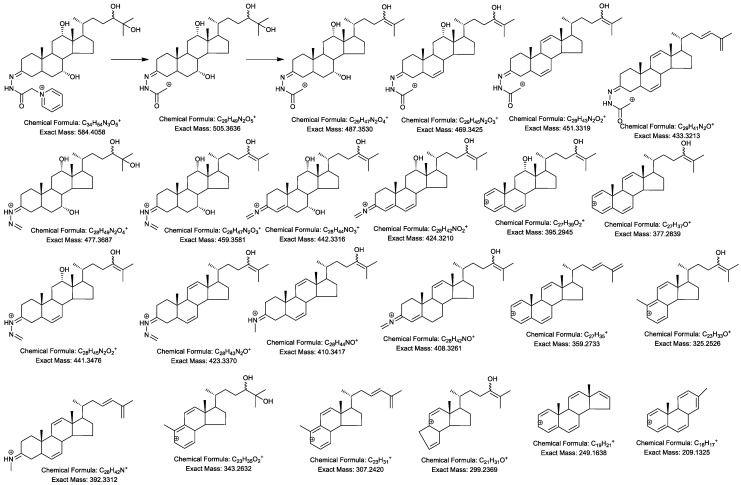
MS^2^ and MS^3^ fragmentation of oxidized/GP-derivatized pentahydroxy-bile alcohols as illustrated by C-3α,7α,12α,24,25-pentol. Fragment ions with a 24-hydroxy-24-ene structure are likely to rearrange to the 24-ketone. For simplicity the fragmented Girard derivatizing group is shown in its linear isomeric form. Cyclic isomers are similar to those depicted in Scheme 1. See Supplemental Schemes S5–S7 for fragmentation schemes of other pentahydroxy-bile alcohols.

**Table 1:** Composition of fragment ions generated from oxidised/GP-derivatised C_27_ bile alcohols and C_27_ and C_24_ acids.

Ion	Composition	C-triol	[^2^H_7_]C-triol	C-tetrol	C-pentol	CA-triol	BA-triol
		*m*/*z*	*m*/*z*	*m*/*z*	*m*/*z*	*m*/*z*	*m*/*z*
[M]^+^	[M]^+^	552.4160	559.4599	568.4109	584.4058	582.3901	540.3432
[M-79]^+^	[M-Py]^+^	473.3738	480.4177	489.3687	505.3636	503.3479	461.3010
[M-79-18]^+^	[M-Py-H_2_O]^+^	455.3632	462.4071	471.3581	487.3530	485.3373	443.2904
[M-79-28]^+^	[M-Py-CO]^+^	445.3789	452.4228	461.3738	477.3687	475.3530	433.3061
[M-79-36]^+^	[M-Py-(H_2_O)_2_]^+^	437.3526	444.3965	453.3475	469.3424	467.3267	425.2798
[M-79-28-18]^+^	[M-Py-CO-H_2_O]^+^	427.3683	434.4122	443.3632	459.3581	457.3424	415.2955
[M-79-36-18]^+^	[M-Py-(H_2_O)_3_]^+^	419.3421	426.3860	435.3370	451.3319	449.3162	407.2693
[M-79-28-18-15]^+^	[M-Py-CO-H_2_O-NH]^+^	412.3574	419.4013	428.3523	444.3472	442.3315	400.2846
[M-79-28-18-17]^+^	[M-Py-CO-H_2_O-NH_3_]^+^	410.3417	417.3856	426.3366	442.3315	440.3158	398.2689
[M-79-28-36]^+^	[M-Py-CO-(H_2_O)_2_]^+^	409.3577	416.4016	425.3526	441.3475	439.3318	397.2849
[M-79-36-36]^+^	[M-Py-(H_2_O)_4_]^+^	401.3315	408.3754	417.3264	433.3213	431.3056	389.2587
[M-79-28-15-31]^+^	[M-Py-CO-NH-CH_2_NH_3_]^+^	399.3258	406.3697	415.3207	431.3156	429.2999	387.2530
[M-79-36-36-2]^+^	[M-Py-(H_2_O)_4_-H_2_]^+^	399.3159	406.3598	415.3108	431.3057	429.2900	387.2431
[M-79-28-36-15]^+^	[M-Py-CO-(H_2_O)_2_-NH]^+^	394.3468	401.3907	410.3417	426.3366	424.3209	382.2740
[M-79-28-36-17]^+^	[M-Py-CO-(H_2_O)_2_-NH_3_]^+^	392.3312	399.3751	408.3261	424.3210	422.3053	380.2584
[M-79-28-36-18]^+^	[M-Py-CO-(H_2_O)_3_]^+^	391.3472	398.3911	407.3421	423.3370	421.3213	379.2744
[M-79-28-18-15-31]^+^	[M-Py-CO-H_2_O-NH-CH_2_NH_3_]^+^	381.3152	388.3591	397.3101	413.3050	411.2893	369.2424
[M-79-36-36-18-2]^+^	[M-Py-(H_2_O)_5_-H_2_]^+^	381.3053	388.3492	397.3002	413.2951	411.2794	369.2325
[M-79-28-36-15-18+2]^+^	[M-Py-CO-(H_2_O)_3_-NH+H_2_]^+^	378.3519	385.3958	394.3468	410.3417	408.3260	366.2791
[M-79-28-36-15-18]^+^	[M-Py-CO-(H_2_O)_3_-NH]^+^	376.3363	383.3802	392.3312	408.3261	406.3104	364.2635
[M-79-28-36-17-18]^+^	[M-Py-CO-(H_2_O)_3_-NH_3_]^+^	374.3206	381.3645	390.3155	406.3104	404.2947	362.2478
[M-79-28-36-15-31]^+^	[M-Py-CO-(H_2_O)_2_-NH-CH_2_NH_3_]^+^	363.3046	370.3485	379.2995	395.2944	393.2787	351.2318
[M-79-28-36-15-36+2]^+^	[M-Py-CO-(H_2_O)_4_-NH+H_2_]^+^	360.3414	367.3853	376.3363	392.3312	390.3155	348.2686
[M-79-28-36-15-31-18]^+^	[M-Py-CO-(H_2_O)_3_-NH-CH_2_NH_3_]^+^	345.2941	352.3380	361.2890	377.2839	375.2682	333.2213
M-79-28-36-15-31-36]^+^	[M-Py-CO-(H_2_O)_4_-NH-CH_2_NH_3_]^+^	327.2835	334.3274	343.2784	359.2733	357.2576	315.2107
[A_3_+H-36]^+^	[A_3_+H-(H_2_O)_2_]^+^	313.2890	320.3329	329.2839	345.2788	343.2631	301.2162
[A_3_-H-36]^+^	[A_3_-H-(H_2_O)_2_]^+^	311.2733	318.3172	327.2682	343.2631	341.2474	299.2005
[A_3_-H-36-18]^+^	[A_3_-H-(H_2_O)_3_]^+^	293.2628	300.3067	309.2577	325.2526	323.2369	281.1900
[A_3_-H-26]^+^		285.2577	292.3016	301.2526	317.2475	315.2318	273.1849
[A_3_-H-36-36]^+^	[A_3_-H-(H_2_O)_4_]^+^	275.2522	282.2961	291.2471	307.2420	305.2263	263.1794
[ABCD-Δx5-H]^+^		249.1638	249.1638	249.1638	249.1638	249.1638	249.1638
[ABC-Δx5-H]^+^		209.1325	209.1325	209.1325	209.1325	209.1325	209.1325

Note: *m*/*z* values are calculated for the chemical compositions. ABCD corresponds to the intact ring structure including C-19 and C-18. ABC corresponds to the intact ring structure including C-19. Δx5 corresponds to 5 double bonds. The fragmenation-route A_3_ is depicted in the inset to Scheme 3. Colour Code: Red, loss of H_2_O; green, loss of CO; blue, loss of NH or NH_3_; purple, loss of CH_2_NH_3_.

## Supplementary Materials

The following are available online. Supplemental Figure S1. MS^2^ ([M]^+^→) spectra of (a) C-3α,7α,12α-triol and (b) C-3α,7α,12α,26-tetrol, and (c) MS^3^ ([M]^+^→[M-Py-18]^+^→) spectrum of C-3α,7α,12α,26-tetrol. Data were generated on the Orbitrap-Elite mass spectrometer as in Figure 1. Supplemental Figure S2. Mass spectra of oxidized/GP-derivatized bile alcohols and acids recorded at the peak of their chromatographic elution. Except for C-3α,7α,12α,26-tetrol, C-3α,7α,12α,25,26-pentol, C-3α,7α,12α,26,27-pentol and C-3α,7α,12α,23,25-pentol which were injected (35 µL) on-column at 0.2 pg/µL all other analytes were inject on-column at 2 pg/µL. (a) C-3α,7α,12α-triol, (b) C-3α,7α,12α,26-tetrol, (c) CA-3α,7α,12α-triol, (d) C-3α,7α,12α,25-tetrol, (e) [^2^H_7_]C-3β,5α,6β-triol, (f) BA-3β,5α,6β-triol, (g) C-3α,7α,12α,24R,25-pentol, (h) C-3α,7α,12α,24S,25-pentol, (i) C-3α,7α,12α,25,26-pentol, (j) C-3α,7α,12α,26,27-pentol and (k) C-3α,7α,12α,23,25-pentol. Data were generated on the Orbitrap-Elite mass spectrometer as in Figure 1. Scheme S1: MS^2^ and MS^3^ fragmentation of oxidized/GP derivatized C-3α,7α,12α,26-tetrol. For simplicity the fragmented Girard derivatizing group is shown in its linear isomeric form. Cyclic forms are depicted in Scheme 1. Scheme S2: MS^2^ and MS^3^ fragmentation of oxidized/GP derivatized CA-3α,7α,12α-triol. For simplicity the fragmented Girard derivatizing group is shown in its linear isomeric form. Cyclic forms are depicted in Scheme 1. Scheme S3: MS^2^ and MS^3^ fragmentation of oxidized/GP derivatized C-3α,7α,12α,25-tetrol. For simplicity the fragmented Girard derivatizing group is shown in its linear isomeric form. Cyclic forms are depicted in Scheme 1. Scheme S4: MS^2^ and MS^3^ fragmentation of oxidized/GP derivatized BA-3α,7α,12α-triol. For simplicity the fragmented Girard derivatizing group is shown in its linear isomeric form. Cyclic forms are depicted in Scheme 1. Scheme S5: MS^2^ and MS^3^ fragmentation of oxidized/GP derivatized C-3α,7α,12α,25,26-pentol. For simplicity the fragmented Girard derivatizing group is shown in its linear isomeric form. Cyclic forms are depicted in Scheme 1. Scheme S6: MS^2^ and MS^3^ fragmentation of oxidized/GP derivatized C-3α,7α,12α,26,27-pentol. For simplicity the fragmented Girard derivatizing group is shown in its linear isomeric form. Cyclic forms are depicted in Scheme 1. Scheme S7: MS^2^ and MS^3^ fragmentation of oxidized/GP derivatized C-3α,7α,12α,23,25-pentol. For simplicity the fragmented Girard derivatizing group is shown in its linear isomeric form. Cyclic forms are depicted in Scheme 1.
