# Supplementary material for: Developing an Enzyme-Assisted Derivatization Method for Analysis of C27 Bile Alcohols and Acids by Electrospray Ionization-Mass Spectrometry
Source: Molecules. 2019 Feb 7;24(3):597. doi: 10.3390/molecules24030597 (PMC6384595; doi:10.3390/molecules24030597)

Supplementary Figures and Schemes

S1a

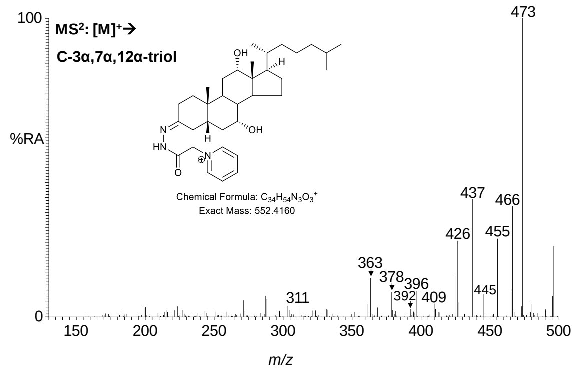
MS²: [M]⁺→
C-3α,7α,12α-triol
Chemical Formula: C34H54N3O3+
Exact Mass: 552.4160
%RA
100
0
473
437
466
426
455
363
378
396
392
409
445
311
150 200 250 300 350 400 450 500
m/z

S1b

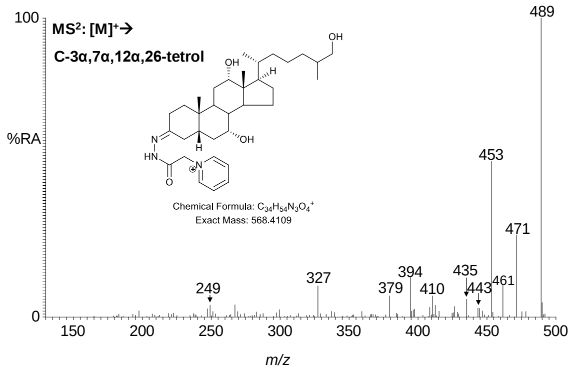
MS²: [M]⁺→
C-3α,7α,12α,26-tetrol
Chemical Formula: C34H54N3O4+
Exact Mass: 568.4109
%RA
100
0
489
453
471
327
394
435
379
410
443
461
249
150 200 250 300 350 400 450 500
m/z

S1c

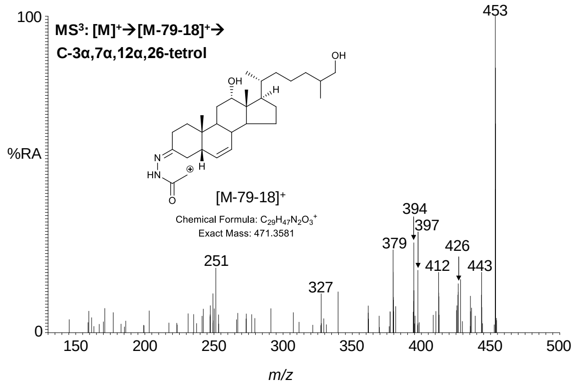
MS³: [M]⁺→[M-79-18]⁺→
C-3α,7α,12α,26-tetrol
[M-79-18]⁺
Chemical Formula: C29H47N2O3+
Exact Mass: 471.3581
%RA
100
0
453
394
397
379
426
412
443
251
327
150 200 250 300 350 400 450 500
m/z

Scheme S1

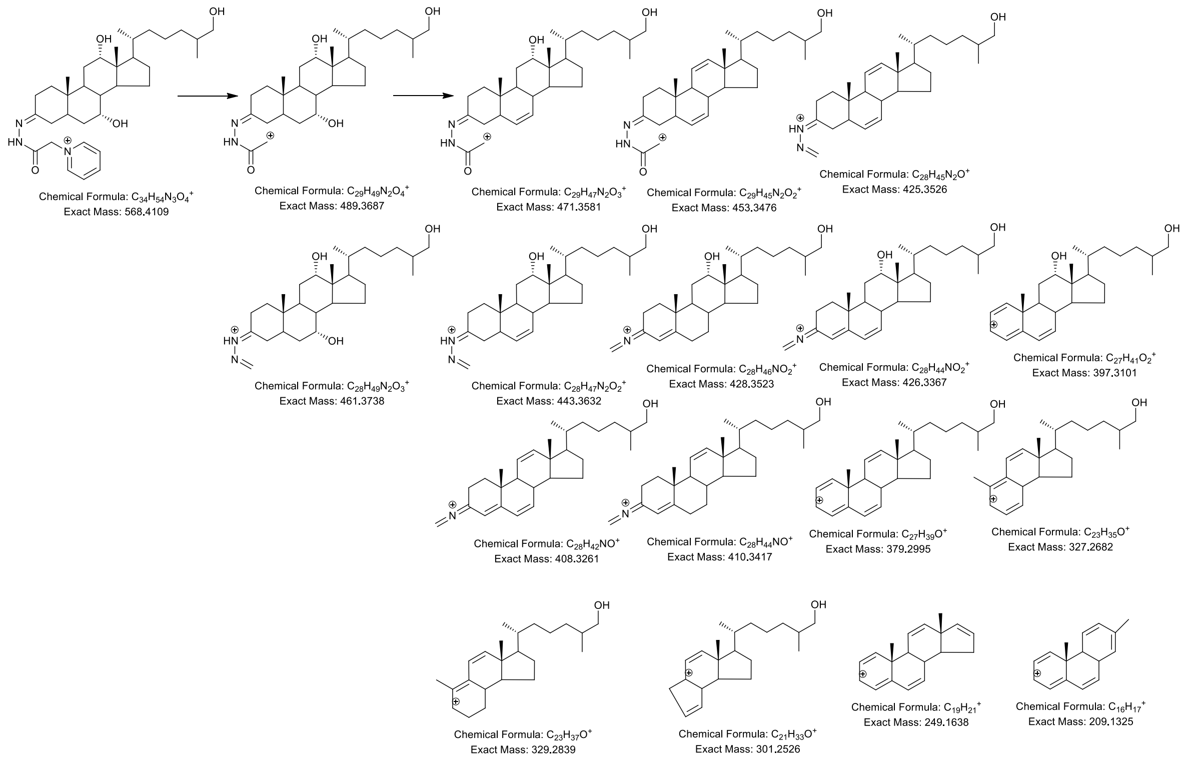

Scheme S2

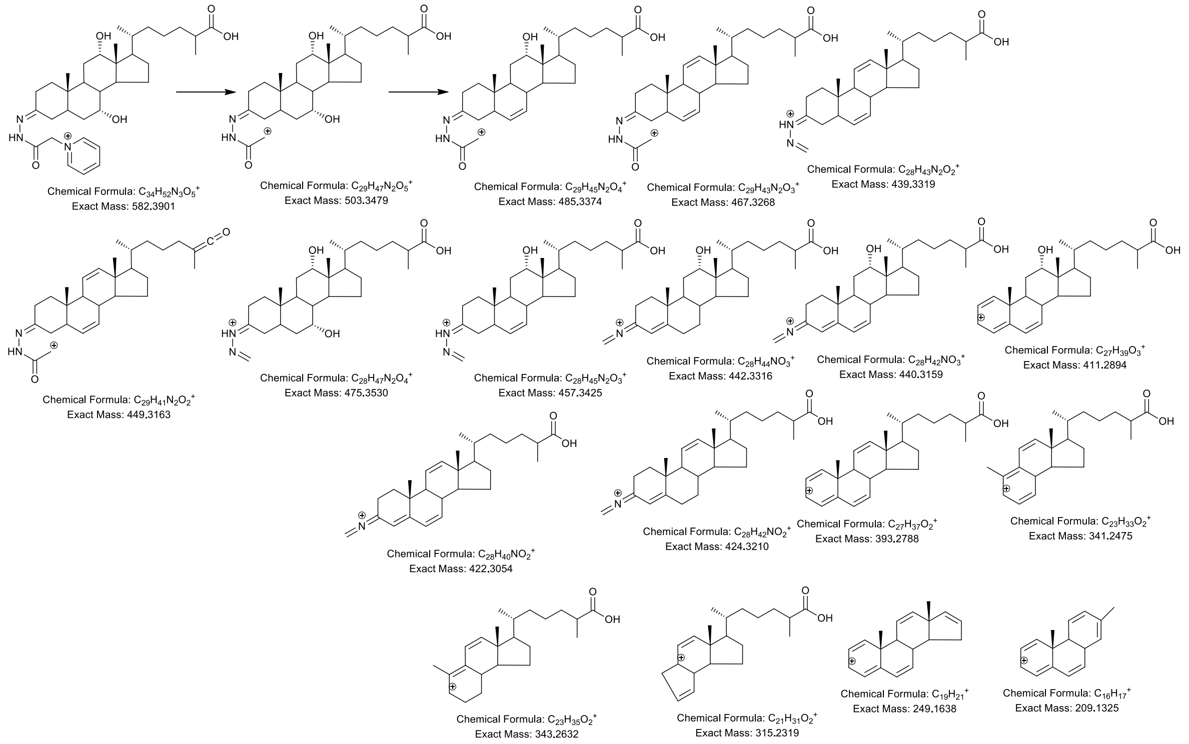

Scheme S3

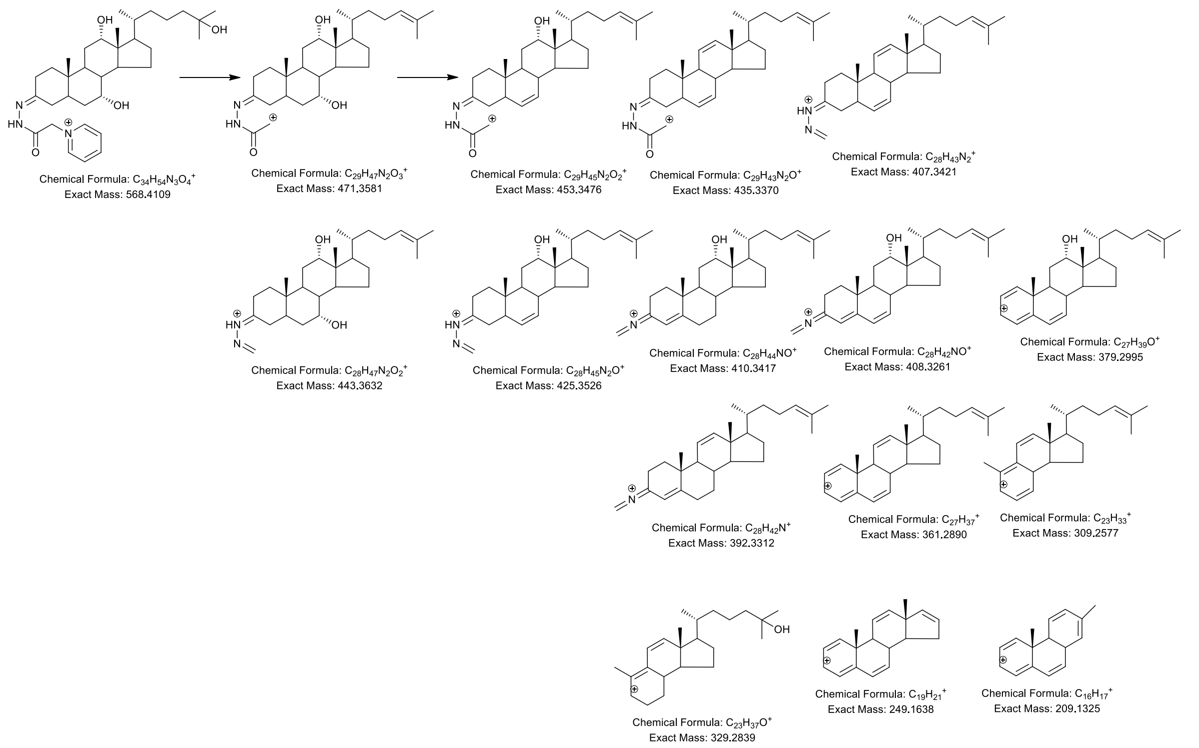

Scheme S4

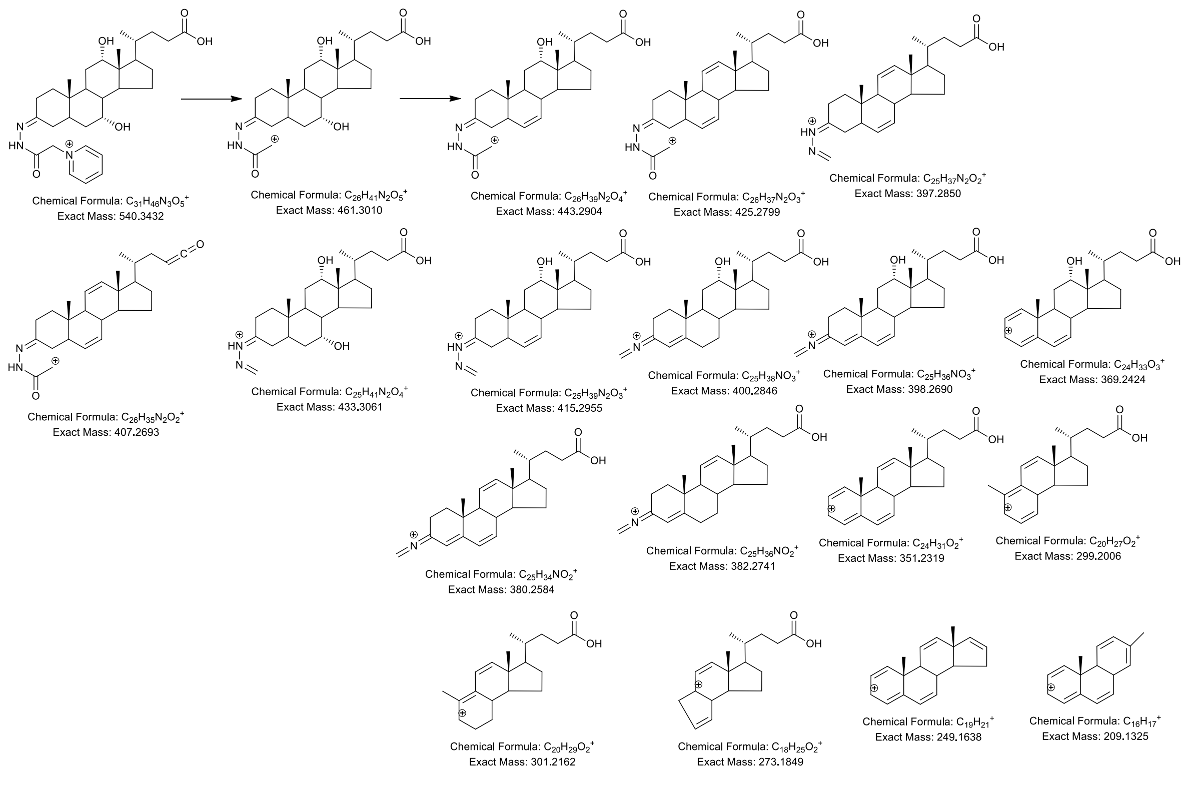

Scheme S5

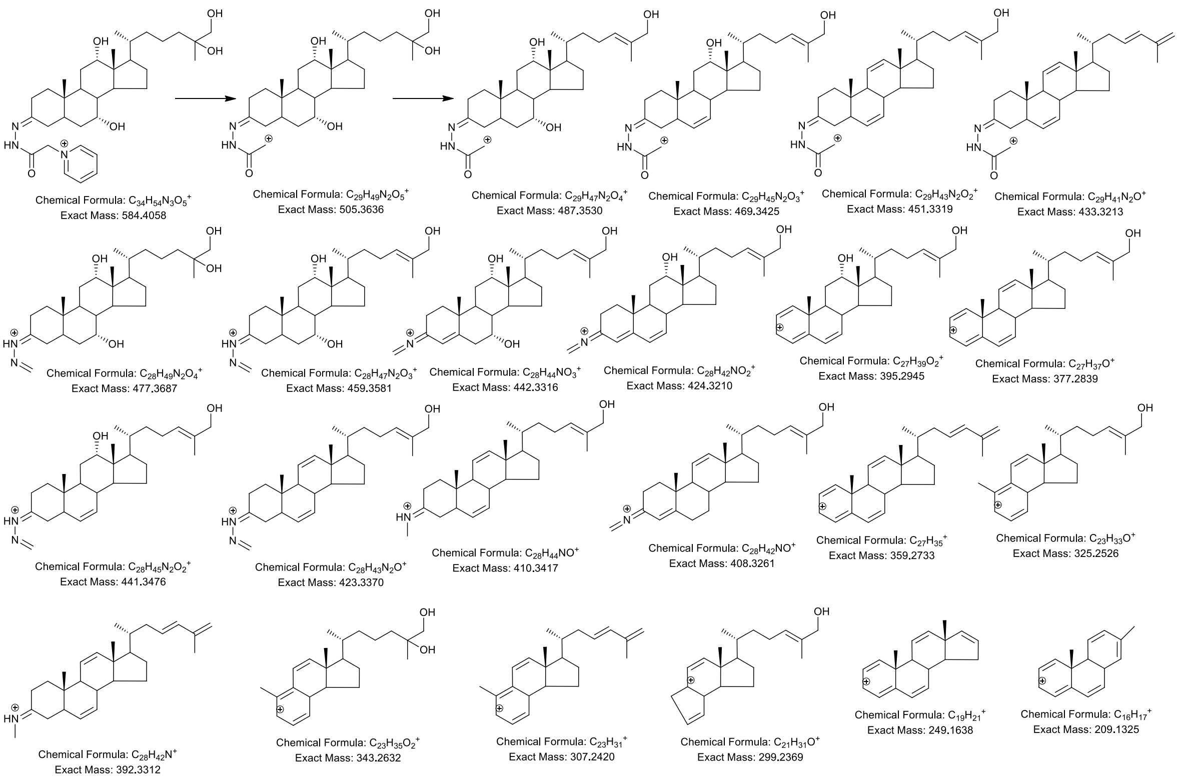

Scheme S6

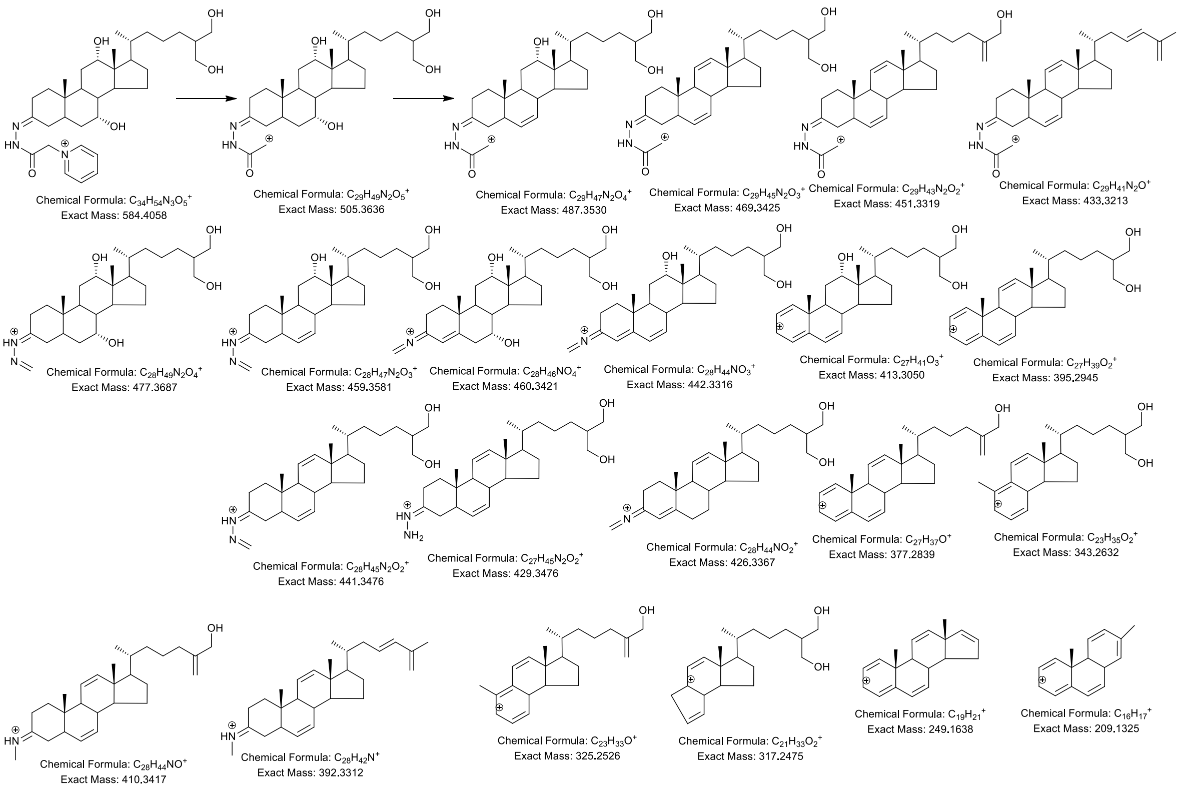

Scheme S7a

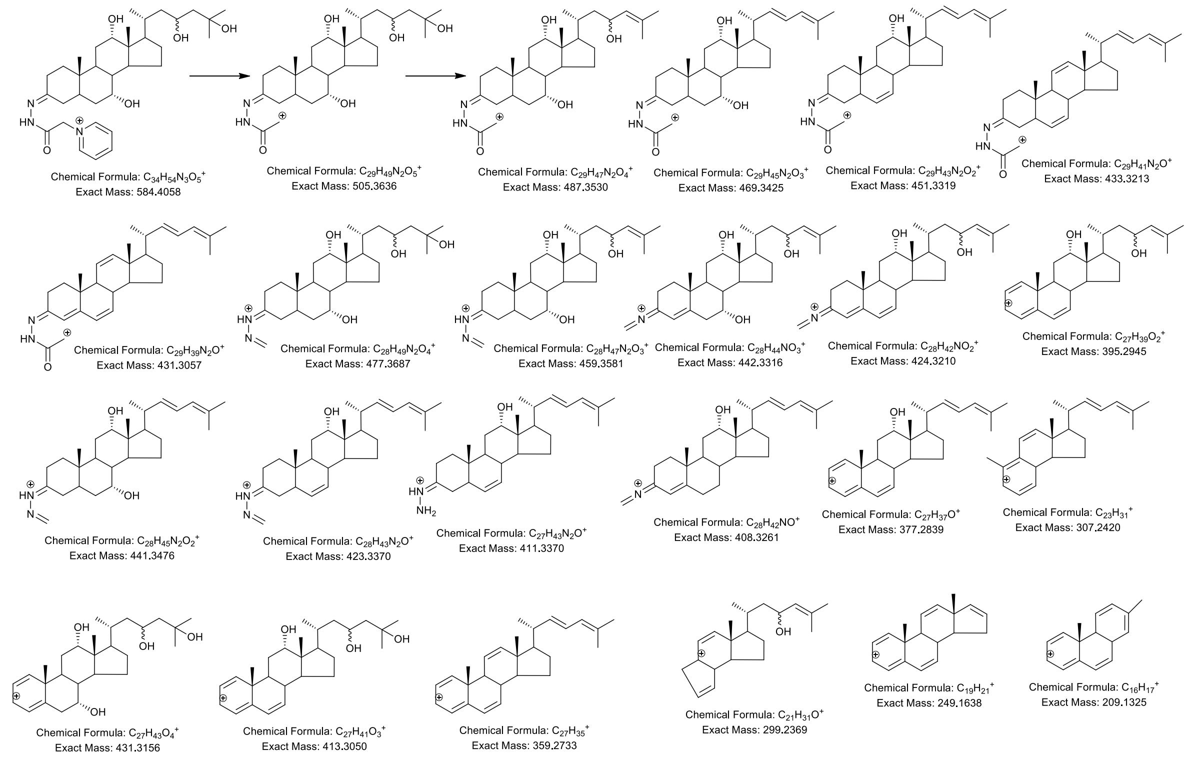

Scheme S7b

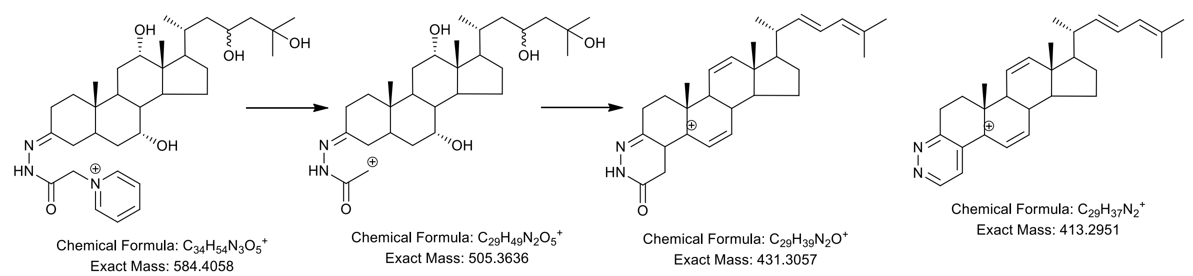

Supplement: Supplementary file 1 [file molecules-24-00597-s001.pdf]
